# Retrospective Comparison of Postoperative Fascia Iliaca Block and Multimodal Drug Injection on Early Function of the Knee in Femoral Fractures Using Retrograde Intramedullary Nailing

**DOI:** 10.1155/2022/7027637

**Published:** 2022-03-19

**Authors:** Songtao Li, Ping Luo, Yuhu Huang, Huarong Xia, Wushu Wei, Wendun Wei, Tianyu Xia, Kai Xu

**Affiliations:** ^1^Department of Orthopedics, No. 924 Hospital of PLA, Guilin, China; ^2^Guangxi Key laboratory of Metabolic Diseases Research, Guilin, China

## Abstract

**Introduction:**

There is a common concern about the pain and rehabilitation of the knee after femoral retrograde intramedullary nailing. It is essential for early postoperative knee function required for physical self-maintenance in daily life. And a favorable rehabilitation of the knee usually promotes the quality of life. However, early rehabilitation is absent or insufficient for many patients in postoperative management. This retrospective study aims to evaluate the effect of early knee function improvement in comparison to postoperative fascia iliaca blocking (FIB) and multimodal drug injection (MDI). *Patients and Methods*. A retrospective analysis of 41 patients receiving femoral fracture treatment with retrograde intramedullary nailing, was performed during 2018–2020. 19 patients were treated with MDI as postoperative analgesia, and 22 patients were treated with FIB. Rehabilitation started on the first postoperative day and lasted for 3 months. Visual analog scale (VAS), the range of motion (ROM) of the knee, and single assessment numeric evaluation (SANE) were assessed.

**Results:**

There was no significant difference shown in any of the demographic, fracture types, and operative time. All patients performed regular and voluntary knee rehabilitation and weight-bearing at home following the instruction from the orthopedic staff. Pain in the FIB group at postoperative 1-day was milder (1.7 ± 1.1), compared with that in the MDI group (2.8 ± 1.3, *p*=0.038). There was a significant difference in VAS between two groups at postoperative 1-month (*p*=0.031), with a peak score in the FIB group (3.3 ± 0.9). At postoperative 3-month, both groups had pain relief with similar VAS (*p*=0.465). The ROM of the knee in both groups was continuously improved during the first three months. The SANE in the MDI group was significantly different compared with FIB at 1-month (*p*=0.026). However, scores of SANE were similar in both groups at 3 months (*p*=0.541). All patients were identified as fractures union at 9-month or 12-month follow-up.

**Conclusion:**

The knee pain was commonly experienced in this series of retrograde femoral nailings. Both MDI and FIB provided immediate and effective pain control after femoral fracture surgery. MDI was more beneficial to continuous pain control and knee rehabilitation in the early follow-up. The extent of pain relief and knee function improvement reached the same level at postoperative 3-month.

## 1. Introduction

Intramedullary nailing is an effective method for femoral shaft fractures. In consideration of patients' conditions such as fractures in the distal femur, obesity, ipsilateral pelvic or hip fracture, or ipsilateral tibia fracture, the use of retrograde intramedullary nailing for femoral shaft fractures has become an attractive practice over the last decades [1–5]. However, with the entry point for retrograde nailing being within the knee, this technique may cause complaints about postoperative pain in the knee [2, 6]. Most of the studies revealed a higher rate of anterior knee pain that is more related with retrograde nailing than antegrade nailing for femoral fractures [2, 7–10]. Moreover, this pain is often exacerbated by walking, kneeling, squatting, and stair climbing. Knee function is another focus in follow-up. It seems that there is no significant difference in knee function in the majority of reports [2, 8–10]. However, some studies found that worse knee scores and range of motion were associated with retrograde nailing [11]. Notably, the knee function in most studies was evaluated in a long-term follow-up, usually at least 1 year. The early rehabilitation of knee function seems to be ignored. The ROM is a basic indicator for knee function, for example, walking requires 67°of ROM, up- and down-stairs require 80°, and sitting in a chair requires 93°. Obviously, early postoperative knee function is an essential requirement for physical self-maintenance of daily living. A favorable functional knee rehabilitation usually promotes the life quality. Furthermore, knee function at 1 month postoperatively may indicate the likelihood of achieving the clinical goal at 12 months [12]. The changes in the knee range of flexion plateaued 3 months after total knee arthroplasty [13]. And poor knee function after knee arthroplasty can be detected through ROM data in the first few weeks [14]. Therefore, it is worthy to pay close attention to early knee rehabilitation after retrograde nailing.

A standard procedure of knee functional rehabilitation usually starts with a continuous passive motion (CPM) on the first day postoperation. Then, the active motion of the knee and weight-bearing exercise are encouraged depending on the tolerance of patients. Usually, the early knee functional rehabilitation is followed by the instructions of physical therapists. Nevertheless, early rehabilitation is absent or insufficient for many patients due to financial difficulty or a lack of home nursing. The confidence and willingness of rehabilitation in early postoperative stage were weakened due to the early pain, although orthopedic staff introduced exercise advise during hospital stay.

The authors speculated that satisfactory postoperative analgesia could facilitate the rehabilitation of the early postoperative stage and improve the knee function in retrograde nailing cases. A multimodal drug periarticular injection was found to relieve pain effectively and promote a better functional recovery among patients receiving total joint replacement [15]. Femoral nerve block has a similar analgesic effect on multimodal periarticular soft tissue injection after total knee arthroplasty as well [16]. This retrospective study is conducted to evaluate the effect of early knee function improvement in comparison with postoperative fascia iliaca blocking with multimodal drug injection.

## 2. Patients and Methods

### 2.1. Patients Information

**Figure 1:**
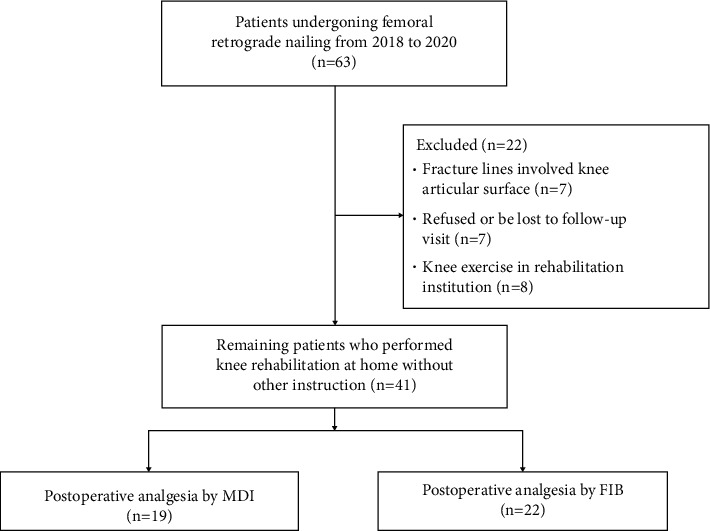
Enrollment flow diagram.

Our institutional Ethical Review Committee reviewed and approved the study protocol. During April 2018 and November 2020, 63 patients suffering from femoral fractures were treated with retrograde intramedullary nailing (Figure 1). 3 distal femoral fractures identified as AO/OTA 33C and 4 ipsilateral tibia plateau fractures were excluded. Also 7 patients were excluded who refused or missed follow-up visits and 8 patients who performed regular exercise in rehabilitation institutions. The remaining 41 patients were retrospectively analyzed. 19 patients before April 2019 were treated with multimodal drug injection (MDI), and 22 patients were treated with fascia iliaca block (FIB) thereafter.

### 2.2. Operation

All patients were treated with rearmed retrograde nailing (Type B DFN, Double Medical, Xiamen, China) within an intercondylar notch approach. Through a longitudinal incision medial to the patellar tendon, the entry point was located at the anterior end of Blumensaat's line on the lateral projection, essentially at the top of the intercondylar notch, in line with the femoral shaft. After opening the canal, a threaded guidewire was inserted. The fracture was closed and reduced, and the canal was rearmed. A retrograde nailing was inserted and locked proximally and distally. Length and rotation were controlled by comparing AP knee and hip images to the contralateral side.

### 2.3. Perioperative Analgesia and Care

All femoral fractures were immobilized with skeletal traction, and dezocine was prescribed at the discretion of the residents. 24 hours before the operation, oral imrecoxib of 100 mg per 12 hours was prescribed. All femoral nailing operations were performed under general laryngeal mask airway anesthesia. Immediately postoperatively, the FIB group received 30 ml of 0.75% ropivacaine for an ultrasound-guided fascia iliaca block. The MDI group received a multimodal drug consisting of 10 ml of 0.75% ropivacaine, 1 ml of 40 mg triamcinolone, 1 ml of 30 mg ketorolac tromethamine, 1 ml of 100 mg pethidine hydrochloride, and saline to make up 50 ml in total. 20 ml of the mixture was injected at the fracture site, 10 ml at the subcutaneous fascia around the incision, and the remanent 20 ml was injected intraarticular after the incision was closed.

Rehabilitation was started on the first postoperative day with CPM of the hip and knee joints. The CPM machine was set to range from 40 degrees of knee flexion to full extension, with attempts to increase by 5–10 degrees of flexion to a maximum of 90°, as the patients tolerated. On the third postoperative day, the patients were encouraged to perform straight leg-raising exercises and active flexion of the hips and knees, from a tolerable range followed by a gradual increase. Partial weight bearing with crutches started as soon as the pain became tolerable. All patients were familiar with early knee rehabilitation following the instruction from the orthopedic staff. They were discharged on the seventh to ninth postoperative day and performed knee rehabilitation voluntarily at home without further professional instruction.

### 2.4. Data Collection

For each patient, the authors recorded knee pain in the visual analog scale (VAS) and the range of motion (ROM) of the knee on the first day, 1 week, 1 month, and 3 months postoperatively. Single assessment numeric evaluation (SANE) of the knee was recorded for knee functional recovery. Radiographs were recorded at 1, 3, 6, 9, and 12 months postoperatively. Callus formation on three out of four cortices and fracture line fading in radiographs were considered as signs of fracture union.

### 2.5. Statistical Analyses

Statistical analysis was performed using SPSS 20.0 software. The Chi-square test to identify differences in sex and fracture types. Student's *t*-test was used to identify differences in age, operative time, VAS, ROM, and SANE of the knee. Statistical significance was accepted for *p* values of <0.05.

## 3. Results

Demographics for patients are presented in Table 1. No significant differences were noted in any of the demographics, fracture types, or operative time. All patients reported regular and voluntary knee rehabilitation and weight-bearing at home according to previous instruction.

**Figure 2:**
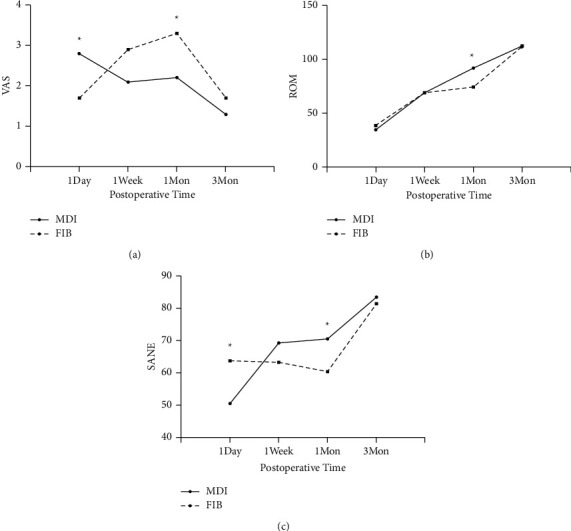
Changes in VAS (a), ROM of knee (b), SANE of knee (c). Data are shown as mean, ^∗^*p* < 0.05.

The comparison of VAS and knee function for the two groups was presented in Figure 2. Knee pain came out in a trend of anesis in the MDI group. Pain in the FIB group at postoperative 1-day was milder (VAS, 1.7 ± 1.1), compared with that in the MDI group (2.8 ± 1.3, *p*=0.038). Then, the VAS of the FIB group ascended to 2.9 ± 1.2 at postoperative 1-week, although there was no significant difference between MDI (2.1 ± 1.1, *p*=0.078). There was a significant difference in VAS between the two groups at postoperative 1-month (*p*=0.031), with a peak score in the FIB group (3.3 ± 0.9). At 3 months, both groups had pain relief with similar VAS (*p*=0.465). ROM of the knee in both groups was continuously improved during early rehabilitation, with the exception of stagnant in the FIB group at 1-week and 1-month (*p*=0.381). And ROM in MDI (92° ± 12°) was better than that in FIB (75° ± 18°, *p*=0.009). SANE of the knee in the FIB group (63.4 ± 9.4) was better than that in MDI (50.4 ± 14.2, *p*=0.012). The comparison between the two groups was reversed at 1-week, although no significant (*p*=0.165). SANE in the MDI group was significantly different compared with FIB at 1-month (*p*=0.026). Finally, both groups got similar scores of SANE at 3-month (*p*=0.541).

**Figure 3:**
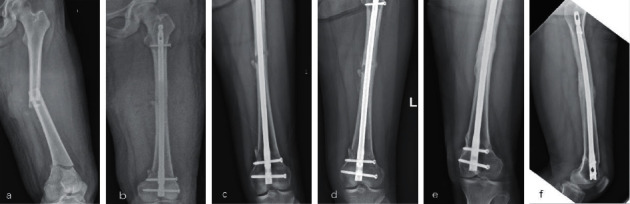
Female, 33, multi-fragment fracture of left femur with AO classification A3 in shaft, and A1 in distal part (a). Treated with retrograde nailing and postoperative FIB, 1-day postoperative radiograph (b). 1 month (c), 3-month (d) follow-up radiographs. 9-month follow-up radiographs indicated union (e, f).

**Figure 4:**
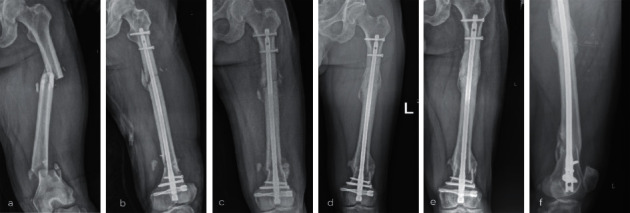
Male, 33, multi-fragment fracture of left femur with AO classification B3 in shaft, and A2 in distal part (a). Treated with retrograde nailing and postoperative MDI, 1-day postoperative radiograph (b). 1 month (c), 3-month (d) follow-up radiographs. 1-year follow-up radiographs indicated union (e, f).

There was no secondary surgery performed such as exchange nailing, bone grafting, or screw removal in present study. All cases are identified as fractures union at 9-month (Figure 3) or 12-month follow-up (Figure 4).

## 4. Discussion

As a part of the golden standard for adult femoral shaft fracture treatment, the retrograde nailing technique is an attractive option available to orthopedists, as is the antegrade nailing technique. A common concern is focused on postoperative knee pain and function due to the introduction through the intercondylar notch of the femur [6, 17, 18]. It is universally accepted that retrograde nailing presents satisfactory results in knee function and pain, both in medium and long-term follow-up [2, 8]. MDI and FIB are popular methods for postoperative pain control after lower extremity surgery [15, 19–21]. However, early rehabilitation of knee function seems to be ignored in many studies. Pain relief and knee function recovery play a significant role in self-care ability, such as wearing socks and shoes, washing feet, going to the toilet, and so on. This study retrospectively compared the effects of MDI and FIB on early pain relief and rehabilitation of the knee after retrograde femoral nailing surgery, specially focused on patients who performed voluntary exercise at home.

Of the 63 cases, we excluded seven cases as having the femoral supracondylar fracture of the AO 33C type or with concomitant ipsilateral tibia plateau fractures. These complicated fractures damage the knee surface and, usually combined with severe soft tissue injury, may deteriorate knee function and cause pain. Eight cases who performed rehabilitation in professional institutions were excluded due to incompatibility with the purpose of this study. SANE is an effective tool for knee function assessment and is friendly to both patients and medical staff [22].

Koehler et al. reported that multimodal drug injections presented immediate pain anesis across a diverse orthopedic trauma population undergoing operative intervention for femoral fractures [21]. The surgical-site injection with a multimodal analgesic cocktail provided the improved pain control at the 4, 8, and 12-hour postoperative time points. Cocktail treatment was more popular in arthroplasty. Multimodal periarticular injection provided comparable analgesia to continuous femoral nerve block after total knee arthroplasty [16]. In our study, the MDI group presented satisfactory pain control during hospital stay and continuously improved pain relief during 3 months of follow-up. The knee function improved in the similar trend.

FIB and femoral nerve block provided similar analgesia after femoral fracture surgery [23]. Femoral nerve blocks for tibial plateau fractures operations demonstrated a similar pain relief compared with patients controlled analgesia (PCA), as reported by Cooke et al. [24]. In our study, the FIB group presented immediate analgesia effects after retrograde nailing and got the same level of pain relief at postoperative 3 months. However, pain rebounded when patients were discharged and performed voluntary knee rehabilitation at home. The knee function improvement came to a standstill at the same time.

The results revealed that FIB provided immediate pain relief rather than long-lasting analgesia. Indeed, the major component for FIB injection was ropivacaine, which provided pain relief for 12–24 hours [25]. Coincidently, most studies concerning FIB or femoral nerve block recorded the score of pain in less than 3 days [20, 26]. The short-term analgesic effect may be one of the explanations for VAS rebounded. Another explanation was the increased active exercise for knee and weight bearing. Angers et al. revealed that femoral nerve block had a negative influence on recovery at 6 weeks and 6 months following total knee replacement [27]. As an important part of knee extensional apparatus, weakened quadriceps may be responsible to the slow rehabilitation of the knee.

For the MDI group, pain and function of the knee displayed continuous relief and improvement. Recent research claimed that intraoperative periarticular injection of multimodal drugs could alleviate the inflammatory response and enhance joint function recovery after hip arthroplasty in elderly patients [19]. Similar multimodal drugs may have equally beneficial effects for the MDI group.

The limitations of our study were attributed to the retrospective analysis and could be underpowered. It was a single center study with a small number of cases, and conclusions could not be generalized. Furthermore, we did not use other scoring systems which were more reliable and comprehensive, such as the Lysholm scale and the American Academy of Orthopedic Surgeons hip and knee scale. The life quality should be quantitatively recorded and analyzed in further study. The correlation of pain and knee function was not quantitatively analyzed. In the future, randomized controlled trials with high quality are needed.

## 5. Conclusion

The knee pain was commonly experienced in this series of retrograde femoral nailings. Both MDI and FIB provided immediate and satisfactory pain control after femoral fracture surgery, but pain rebounded after discharge in the FIB group. MDI was more beneficial to continuously pain control and knee rehabilitation during the first month postoperative. Pain relief and knee function improvement reached the same level at postoperative 3-month.

## Figures and Tables

**Table 1:** Patients information.

Patients information			
Variable	MDI group (*n* = 19)	FIB group (*n* = 22)	*p*
Age, y	43.5 ± 18.1	39.3 ± 17.0	0.557
Sex (M/F)	12/7	16/6	0.737
AO classification			
Femoral shaft	17	21	0.769
A	6	5	
B	6	6	
C	5	8	
Femoral supracondylar	5	6	
A	5	6	
Multi-segmental fractures	3	5	0.703
Operation time (minutes)	145.5 ± 36.1	130.9 ± 31.2	0.291

## Data Availability

The data supporting the findings of the study are available from the corresponding author on reasonable request.
